# Long-lasting Corolla Cultivars in Japanese Azaleas: A Mutant *AP3/DEF* Homolog Identified in Traditional Azalea Cultivars from More Than 300 Years Ago

**DOI:** 10.3389/fpls.2017.02239

**Published:** 2018-01-09

**Authors:** Kyeong-Seong Cheon, Akira Nakatsuka, Keisuke Tasaki, Nobuo Kobayashi

**Affiliations:** ^1^Faculty of Life and Environmental Science, Shimane University, Matsue, Japan; ^2^Genomics Division, National Institute of Agricultural Sciences, Rural Development Administration, Jeonju, South Korea; ^3^Iwate Biotechnology Research Center, Kitakami, Japan

**Keywords:** long-lasting flower, corolla mutant, *AP3/DEF* homolog, transcriptional factors, retrotransposon, evergreen azalea

## Abstract

Floral shape in higher plants typically requires genetic regulation through MADS transcription factors. In Japan, hundreds of azalea cultivars including flower shape mutations have been selected from the diversity of endogenous species and natural hybrids since the early 17th century, the Edo era (1603–1867). The long-lasting trait, known as “Misome-shō” in Japanese, has been identified in several species and cultivar groups of evergreen azaleas (*Rhododendron* L.) from three hundred years ago in Japan. However, the natural mutation conferring the long-lasting trait in azalea remains unknown. Here, we showed MADS-box gene mutations in long-lasting flowers, *R. kaempferi* ‘Nikkō-misome,’ *R. macrosepalum* ‘Kochō-zoroi,’ *R. indicum* ‘Chōjyu-hō,’ and *R.* × *hannoense* ‘Amagi-beni-chōjyu.’ All of the long-lasting flowers exhibited small-sized corollas with stomata during long blooming. In the long-lasting flowers, transcript of the *APETALA3 (AP3)/DEFICIENS (DEF)* homolog was reduced, and an LTR-retrotransposon was independently inserted into exons 1, 2, and 7 or an unknown sequence in exon 1 in gDNA of each cultivar. This insertion apparently abolished the normal mRNA sequence of the *AP3/DEF* homolog in long-lasting flowers. Also, long-lasting flowers were shown from F2 hybrids that had homozygous *ap3/def* alleles. Therefore, we concluded that the loss of function of the *AP3/DEF* homolog through a transposable element insertion may confer a stable long-lasting mutation in evergreen azaleas.

## Introduction

**FIGURE 1:**
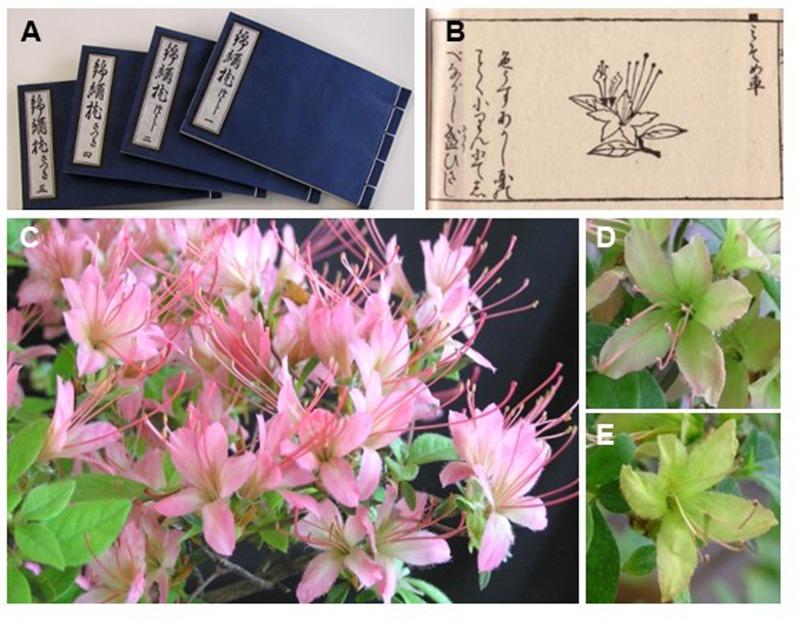
Information of long-lasting trait in Japanese azalea. **(A,B)** Photograph of “Kinshū-makura” **(A)** and ‘Misome-guruma’ **(B)**, edited in 1692. **(C–E)** Flower photos of *Rhododendron kaempferi* ‘Nikkō-misome’ after just flowering **(C)**, approximately 50 days **(D)**, and approximately 100 days **(E)**.

Species of the genus *Rhododendron* (Ericaceae), subgenus *Tsutsusi*, section *Tsutsusi* are important genetic resources for evergreen azalea cultivars used as ornamental shrubs or potted azaleas in many regions worldwide, including Asia, Europe, and America. Japan has many wild evergreen azalea species, and hundreds of azalea cultivars have been selected from natural populations of endogenous azalea species and hybrids since the Edo era (1603–1867) (Yamazaki, 1996). A monograph on azaleas, “Kinshū-makura” (**Figure 1A**), edited in 1692, described more than 300 azalea cultivars, and several of the cultivars described in this monograph still exist and have unique flower and leaf characteristics (Ito, 1984). This monograph provided the first description of ‘Misome-guruma’ (**Figure 1B**), a cultivar possessing small light-red flowers, long stamens, and a long blooming season, with the flowers turning greenish in June (Ito, 1984). The ‘Misome-guruma’ has been defined as “Misome-shō,” a long-lasting trait in Japanese, and “Misome-shō” species have been identified in several azalea cultivars. The long-lasting flower mechanisms remain largely unknown; however, the trait is characterized by a temporal color change and a long-lasting corolla (**Figures 1C–E**) that is smaller than other evergreen azaleas and may be derived from the sepaloid corolla (Kobayashi et al., 2010; Gobara et al., 2017). In genetic analysis by crossing of azaleas, normal flowers were shown from all F1 progenies between normal and long-lasting flowers, and long-lasting flowers were detected from 1/3 F2 progenies. Therefore, long-lasting flower was shown as recessive trait to normal flower and was controlled by single gene (Gobara et al., 2017).

The corolla mutant of long-lasting flowers is likely associated with the ABC model. Flower development has been studied using *Arabidopsis* and *Antirrhinum* as model plants (Coen and Meyerowitz, 1991). In these model plants, floral homeotic mutant have been described converting to other organs. Most of these mutants have been found from different mutant alleles including one or more MADS-box gene encoding putative transcription factors (Weigel and Meyerowitz, 1994). The floral homeotic MADS-box genes have been classified into three classes by different function according to ABC model (Coen and Meyerowitz, 1991; Weigel and Meyerowitz, 1994). In summary, for *Arabidopsis*, the A-class gene *APETALA1* (*AP1*) controls sepal formation in whorl 1; *AP1* and two B-class genes [*APETALA3* (*AP3*) and *PISTILLATA* (*PI*)] together control petal formation in whorl 2; the B-class genes and the C-class gene *AGAMOUS* (*AG*) together control stamen formation in whorl 3; and *AG* alone control carpel formation in whorl 4. The B-class *PI* and *AP3* proteins are functional partners and form a heterodimer for petal formation (Yang et al., 2003). A loss-of-function mutation in either *PI* or *AP3* produces sepals in two outer whorls of flowers (Jack et al., 1992). Also, in case of *Antirrhinum majus*, similar mutant phenotypes are described for mutation of the gene *GLOBOSA* (*GLO*) and/or *DEFICIENS* (*DEF*), homologs to *PI* and *AP3*, respectively (Sommer et al., 1990; Tröbner et al., 1992). In B-class gene mutants, B-class loss-of-function mutants have sepal or sepaloid petal instead of petals in the second whorl in model and horticultural plants (Sommer et al., 1990; Tröbner et al., 1992; Weigel and Meyerowitz, 1994; De Byzova et al., 2004; Vandenbussche et al., 2004; Drea et al., 2007; Hirai et al., 2010; Gong et al., 2017). In B-class genes, the natural corolla mutation of long-lasting azaleas might be associated with the *AP3/DEF* gene, as the sepaloid petals or sepals in whorl 2 were produced from artificial *ap3/def* and/or the *pi/glo* mutant (Vandenbussche et al., 2004), whereas the stamens in whorl 3 were not observed in artificial and natural *pi/glo* mutants in horticulture plants (Yao et al., 2001; Vandenbussche et al., 2004). Thus, we postulated that the long-lasting flower is due to mutation homologs of *AP3/DEF* in the azalea.

In a previous study, we isolated two full-length sequences of *PI/GLO* from *R. obtusum* (Cheon et al., 2016) and a partial length of the *AP3/DEF* homolog from *R. pulchrum* (Cheon et al., 2011) and reported that *PI/GLO* and *AP3/DEF* homologs participated in corolla development in the evergreen azaleas (Tasaki et al., 2012a; Cheon et al., 2016). In this study, to further characterize long-lasting flowers in evergreen azaleas, including *R. kaempferi* ‘Nikkō-misome,’ *R. macrosepalum* ‘Kochō-zoroi,’ *R. indicum* ‘Chōjyu-hō,’ and *R.* × *hannoense* ‘Amagi-beni-chōjyu,’ and to understand how mutant genes result in long-lasting flowers, we investigated the floral morphologies and B-class genes using the cultivars and progenies from a cross between normal and long-lasting flowers.

## Materials and Methods

### Plant Materials

**FIGURE 2:**
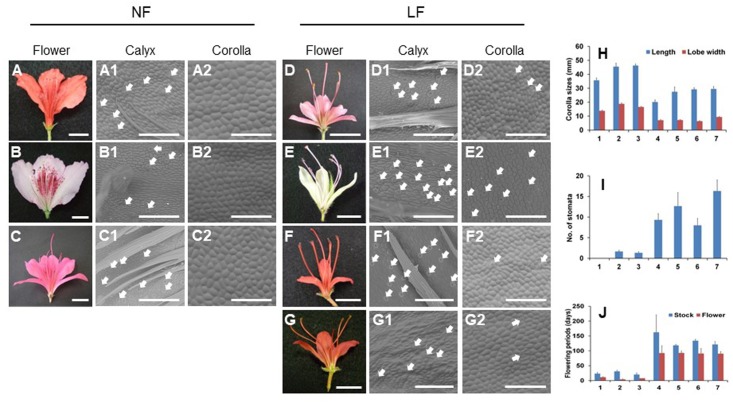
Floral morphological analysis of normal (NF) and long-lasting flowers (LF). **(A–G2)** Photos of flowers **(A–G)** and abaxial epidermal cells of calyx **(A1–G1)** and corollas **(A2–G2)** in normal **(A–C)** and long-lasting flowers **(D–G)**. **(H–J)** Analyses of floral phenotypes, such as corolla sizes (mm) **(H)**, number of stomata **(I)**, and flowering periods (days) **(J)**. The bars for flowers and abaxial epidermal cells indicate 1 cm and 250 μm, respectively. Closed arrows indicate stomata. The error bars indicate the mean ± SE of three biological replicates. The following numbers indicate the azaleas cultivars; 1, *R. kaempferi* **(A–A2)**; 2, *R. macrospalum* **(B–B2)**; 3, *R. indicum* ‘Ōsakazuki’ **(C–C2)**; 4, *R. kaempferi* ‘Nikkō-misome’ **(D–D2)**; 5, *R. macrosepalum* ‘Kochō-zoroi’ **(E–E2)**; 6, *R. indicum* ‘Chōjyu-hō’ **(F–F2)**; 7, *R.* × *hannoense* ‘Amagi-beni-chōjyu’ **(G–G2)**.

The normal-flower azaleas *R. kaempferi* (**Figure 2A**), *R. macrosepalum* (**Figure 2B**), and *R. indicum* ‘Ōsakazuki’ (**Figure 2C**), Kurume hybrid ‘Wakakaede,’ and *R. oldhamii*, the long-lasting flower azaleas *R. kaempferi* ‘Nikkō-misome’ (**Figures 1C**, **2D**), *R. macrosepalum* ‘Kochō-zoroi’ (**Figure 2E**), *R. indicum* ‘Chōjyu-hō’ (**Figure 2F**), and *R.* × *hannoense* ‘Amagi-beni-chōjyu’ (**Figure 2G**), and several progenies between normal and long-lasting flowers used in this study were obtained from the azalea resources collection of the Plant Breeding Laboratory of the Faculty of Life and Environmental Sciences of Shimane University (**Figure 1**). For the extraction of genomic DNA (gDNA) and total RNA, three leaves, ten floral buds, and five flowers were collected from plant materials. These samples were immediately frozen in liquid nitrogen and stored at -80°C until the extraction of gDNAs and total RNAs.

### Flower Morphological Investigation

The length of the corolla and the width of the corolla lobe were measured in 3–10 fully expanded flowers per individual plant (Hang et al., 2010; Gobara et al., 2017). The observation of abaxial epidermal cells was performed using a Scanning Electron Microscope (*HITACHI TM3000*) (Cheon et al., 2016). The stomatal density of abaxial surface in the corolla was determined using Suzuki’s Universal Microprinting Method (*SUMP; SUMP Laboratory*) and a light microscope (*Nikon*) (Tasaki et al., 2012b). The date of flower opening and abscission was investigated in both the stock and the flower (Gobara et al., 2017).

### mRNA Analysis of *AP3/DEF* Homologs

Ten floral buds from each plant and five flowers from wild type and cultivar of *R. kaempferi* were collected and used for RNA extraction, *AP3/DEF* homolog isolation, semi-quantitative reverse transcription-PCR (semi-qRT-PCR), and RT-qPCR. Total RNA was extracted by RNeasy plant mini kit (*Qiagen*) and treated with DNase I (*Promega*) as previously described (Cheon et al., 2011). These RNA samples were subjected to semi-qRT-PCR and RT-qPCR using ReverTra-Ace (*TOYOBO*) according to the manufacturer’s instructions. The *R. kaempferi* total RNA sample and the 5′-Full RACE Core Set (*TaKaRa*) was used for 5′ RACE PCR with the partial sequence of the *AP3/DEF* homolog (Cheon et al., 2011). The primers of full-length *AP3/DEF* homolog, from 5′ untranslated region (UTR) to 3′ UTR, were P1-F (TCTCTTCCTCAACCGAGATCTG) and P1-R (GGTTCTACAACATCACAAGTGC) (**Supplementary Figure S1A**). PCR analysis was used to identify the full-length *AP3/DEF* homolog in the cDNA of *R. kaempferi*. Each 10 μl reaction mixture contained 1 × Ex Taq buffer, 200 μM dNTPs, 0.2 μM of each primer, 1.25 U Ex Taq (*Takara*), and 25 ng cDNA template. The reaction conditions were as follows: preheating at 94°C for 1 min; 35 cycles of denaturation at 94°C for 30 s, annealing at 60°C for 30 s, and extension at 72°C for 30 s; and final extension at 72°C for 1 min. The fragments were cloned into the pGEM-T easy vector (*Promega*), transformed into DH5α (*Nippon gene*) and subsequently sequenced using the BigDye Terminator version 3.1 Cycle Sequencing Kit (*Applied Biosystems*) and an ABI 3130 genetic analyser (*Applied Biosystems*). The deduced amino acid sequences were aligned by the NCBI BLASTp function and Genetyx ver.10 (*Software Development Co.*).

For expression analysis, semi-qRT-PCR was performed for B-class genes. For *AP3/DEF* homolog, each 10 μl reaction mixture contained 1 × Ex Taq buffer, 200 μM dNTPs, 0.2 μM of each primer, 1.25 U Ex-Taq (*Takara*), and 2.5 ng cDNA template. The primer sets were P1-F and P1-R for set 1, P1-F and P2-R for set 2, P3-F and P3-R for set 3, P4-F and P4-R for set 4, and P5-F and P1-R for set 5 (**Supplementary Figure S1A**). The reaction conditions were as follows: preheating at 94°C for 1 min; 35 cycles of denaturation at 94°C for 10 s/30 s, annealing at 61°C or 63°C for 10 s/30 s, and extension at 72°C for 10 s/30 s; and final extension at 72°C for 1 min. Also, expression analysis of *AP3/DEF* and *PI/GLO* homologs in each floral organ was performed according to previous study (Tasaki et al., 2012a). RT-qPCR with one biological and three technical replicates was performed using the Thermal Cycler Dice Real-Time System, SYBR Premix Ex Taq II (*TaKaRa*) and primers (**Supplementary Table S1**). The housekeeping gene *ACTIN* was used to normalize the RT-qPCR output (Cheon et al., 2011).

### Genomic DNA Analysis of the *AP3/DEF* Homolog

gDNAs were extracted from the young leaves of plants using the modified CTAB method (Kobayashi et al., 1998). P-F1 and P-R1 primers were used for *AP3/DEF* homolog isolation in gDNA of *R. kaempferi*. However, because the homolog was not amplified by these primers in each long-lasting cultivar, inverse PCR (Ochman et al., 1988) was performed after digesting *R. kaempferi* gDNA with *Hin*dIII. Finally, the full-sized *AP3/DEF* homolog, including the 5′ upstream region, was isolated from the gDNAs of *R. kaempferi* and long-lasting flowers. Each 10 μl reaction mixture contained 1 × PrimeSTAR GXL buffer, 200 μM dNTPs, 0.2 μM of each primer, 1.25 U PrimeSTAR GXL DNA polymerase, and 25 ng gDNA in *R. kaempferi* or each 25 μl reaction mixture contained 50 ng gDNA template of long-lasting flowers with 5P-F1 (TTCCGACCCAACTCACATAC) and/or 5P-F2 (CGCAAGTCCCAACTCACATA), and P1-R primers. The conditions for *R. kaempferi* and long-lasting flowers were as follows: preheating at 98°C for 10 min; 30–35 cycles of denaturation at 98°C for 10 s, annealing at 60°C for 15 s, and extension at 68°C for 3–12 min; and final extension at 68°C for 15 min. The PCR products were amplified with PrimeSTAR GXL DNA polymerase (*TaKaRa*) and acquired from overhanging dA at the 3′-ends using an A-overhang mixture from the Mighty TA-cloning Reagent Set for PrimeSTAR, followed by sequencing as described above. For analysis of mutant region in azalea *AP3/DEF* sequences, *R. kaempferi* mRNA and gDNA sequences of *AP3/DEF* homologs were used as reference. After exon and intron positions of *AP3/DEF* homologs were analyzed using mRNA and gDNA sequences of *R. kaempferi*, the isolated *AP3/DEF* sequences from gDNAs of *R. kaempferi*, *R. kaempferi* ‘Nikkō-misome,’ *R. macrosepalum* ‘Kochō-zoroi,’ *R. indicum* ‘Chōjyu-hō,’ and *R.* × *hannoense* ‘Amagi-beni-chōjyu’ were aligned by Genetyx ver.10 (*Software Development Co.*). PCR analysis for mutant allele was performed using Ex-taq (*TaKaRa*) and primers (**Supplementary Table S1**).

## Results

### Corolla Mutations Develop to Long-lasting Flowers

To analyze the floral phenotype of long-lasting flowers, we investigated several corolla morphologies (**Figure 2**). Each floral organ of long-lasting flowers existed in each whorl as normal flowers (**Figures 2A–G**). The abaxial epidermal cells of corollas in long-lasting flowers were smaller compared with normal flowers and were similar to the abaxial epidermal cells of calyx (**Figures 2A1–G2**). However, the length and lobe width of the corollas in long-lasting flowers were also smaller compared with normal flowers (**Figure 2H**), and a higher number of stomata were observed on the abaxial surface of the corollas in long-lasting flowers compared with normal flowers (**Figures 2A2–G2,I**). The flowering periods, which facilitate corolla maintenance after flowering, were longer in long-lasting flowers than in normal flowers (**Figure 2J**). Thus, based on these observations of floral morphologies, we confirmed that long-lasting flowers have small-sized corollas with stomata during long blooming period and that the corollas of long-lasting flowers show the conversion of normal corollas to sepaloid corollas, whereas the stamens remain unaffected.

### Isolation of *AP3/DEF* Homolog from *R. kaempferi*

We cloned the azalea *AP3/DEF* homolog using a PCR approach. Based on a partial *RpAP3* sequence, the region from MADS domain to 3′ UTRs, in previously study (Cheon et al., 2011), the 5′ UTR were further amplified by using two nested PCR primers (**Supplementary Table S1**). Ten clones containing the 5′ UTR fragments were sequenced. Subsequently, when we cloned the azalea *AP3/DEF* homolog by using a PCR approach with a P1 primer set, two clones were detected from *R. kaempferi* (**Supplementary Figure S1A**). These cloned mRNA sequences involved a MADS-domain, a K-box, and the C-terminal region (Sommer et al., 1990); and showed 76% amino sequence identity with the DEF genes of *A. majus* (**Supplementary Figure S1B**). The putative azalea *AP3/DEF* homologs were named *RkAP3a* (approximately 824 bp, DDBJ accession number AB853117) and *RkAP3b* (approximately 826 bp, DDBJ Acc. No. AB853118), respectively.

### Abnormal Sequences of *AP3/DEF* Homologs Affected to Reduce Expression in Long-lasting Flowers

**FIGURE 3:**
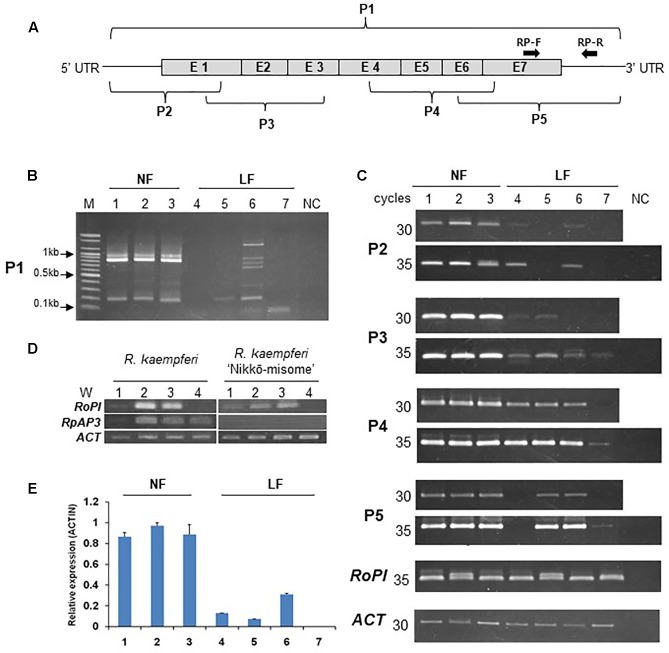
Expression and structure analysis of the *AP3/DEF* homolog in the cDNA of floral buds and organs in normal (NF) and long-lasting flowers (LF). **(A)** Schematic diagram of the *AP3/DEF* homolog sequences according to **Supplementary Figure S1A**. P1–5 and RP-F and -R indicate primer set in this study. **(B,C)** Analysis of PCR amplification using semi-qRT-PCR; analysis of *AP3/DEF* homolog using P1-F and -R primer set **(B)** and other primers **(C)**. M and NC indicate 100 bp DNA ladder (*Bioneer*) and negative control, respectively. **(D)** Expression analysis of *RoPI* and *RkAP3* genes using semi-qRT-PCR in floral organs of *R. kaempferi* and its cultivar ‘Nikkō-misome.’ W1–4 indicate calyx, corolla, stamens, and carpels, respectively. **(E)** Expression analysis of the *AP3/DEF* homolog using RT-qPCR. The error bars indicate the mean ± SE of three technical replicates. The numbers correspond to the plant materials; normal flowers *R. kaempferi* (1), *R. macrospalum* (2), and *R. indicum* ‘Ōsakazuki’ (3) and long-lasting flowers *R. kaempferi* ‘Nikkō-misome’ (4), *R. macrosepalum* ‘Kochō-zoroi’ (5), *R. indicum* ‘Chōjyu-hō’ (6), and *R.* × *hannoense* ‘Amagi-beni-chōjyu’ (7).

**FIGURE 4:**
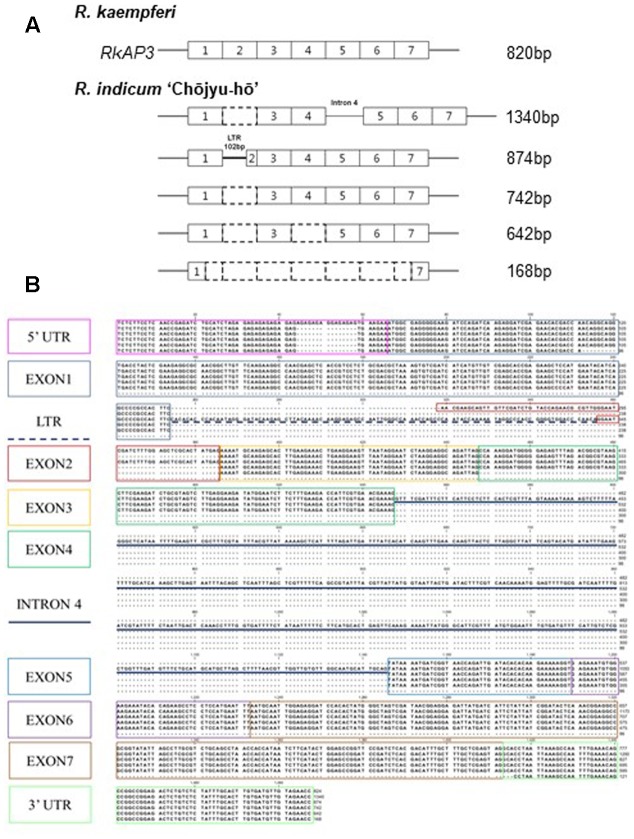
Sequence analysis of the *AP3/DEF* homolog transcript variants in *R. indicum* ‘Chōjyu-hō’ (**Figure 3B**). **(A)** Schematic diagrams of *AP3/DEF* homolog sequences in *R. indicum* ‘Chōjyu-hō.’ **(B)** Sequences of transcript variants. The colored boxes indicate the region of mRNA sequences corresponding to the same color lines.

PCR analyses were carried out to investigate the mRNA structure and expression level of the azalea *AP3/DEF* and *PI/GLO* homolog in floral buds and organs (**Figure 3**). In PCR products of primer set 1, P1-F and P1-R (**Figure 3A** and **Supplementary Figure S1A**), the PCR product predicted for the full-size sequence (approximately 800-bp) was detected from all normal flowers (**Figure 3B**). However, the normal sized band was not detected from long-lasting flowers and multi-bands were detected from *R. indicum* ‘Chōjyu-hō’ in especially (**Figure 3B**). When we analyzed the sequences of multi bands, insertion and deletion of sequences were detected from five clones, which have 1340-, 874-, 742-, 642-, and 168-bp sizes, of *AP3/DEF* homolog in *R. indicum* ‘Chōjyu-hō’ (**Figure 4**). Moreover, PCR analysis was carried out to investigate why the full-sized *AP3/DEF* homolog was not amplified (**Figure 3C**). The amplified PCR product (approximately 250 bp) of primer set 2, P1-F and P2-R, was not detected from *R. macrosepalum* ‘Kochō-zoroi’ or *R.* × *hannoense* ‘Amagi-beni-chōjyu,’ and the PCR product (approximately 135 bp) expression of primer set 3, P3-F and P3-R, in long-lasting flowers were lower than in normal flowers. The PCR product (approximately 260 bp) of primer set 4, P4-F and P4-R, was detected from the cDNA of floral buds in all plant materials. The PCR product (approximately 284 bp) of primer set 5, P5-F and P1-R, was not detected from *R. kaempferi* ‘Nikkō-misome.’ In floral organs, *PI/GLO* homolog was mainly expressed from corolla and stamen in both normal and long-lasting flowers, whereas *AP3/DEF* homolog was not expressed from all floral organs in long-lasting flowers (**Figure 3D**). In addition to semi-qRT-PCR, the results of RT-qPCR showed that the expression of the *AP3/DEF* homolog in floral buds of long-lasting cultivars was lower than that in floral buds of normal cultivars (**Figure 3E**).

### Insertion Mutation Detected in *AP3/DEF* Homolog of Long-lasting Flowers

**FIGURE 5:**
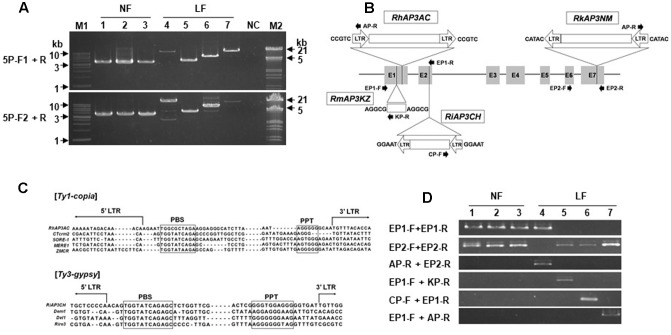
The *AP3/DEF* homolog analysis in gDNA from normal (NF) and long-lasting flowers (LF). **(A)** gDNA fragments were amplified from normal and long-lasting flowers by using primers 5P-F1, 5P-F2, and P1-R. M1, M2, and NC indicate hyperladder1 kb (*Bioline*) and lambda DNA/*Eco*RI+*Hin*dIII conventional DNA digest marker (*Promega*), and negative control, respectively. **(B)** Schematic diagram of the structures of the *AP3/DEF* homolog in long-lasting flowers. E1–7 and lines indicate each exon and each intron, respectively. The open arrows and boxes indicate insertions of TEs. The five nucleotides indicate TSD sequences. The closed arrows indicate primers for TE analysis. **(C)** Comparison among the sequences of LTR-retrotransposons. The boxes indicate the sequences of the PBS and PPT regions. The names of LTR-retrotransposons included *RhAP3AC*, *Rhododendron* × *hannoense* ‘Amagi-beni-chōjyu’; *CTerm2*, *Malus* × *domestica*; *SORE-1*, *Glycine max*; *MERE1*, *Medicago truncatula*; *ZMCR*, *Zea mays*; *RiAP3CH*, *R. indicum* ‘Chōjyu-hō’; *Dem1*, *Malus* × *domestica*; *Del1*, *Lilium henryi*; and *Rire3*, *Oryza sativa*. **(D)** PCR analysis for TE confirmation. Normal flowers *R. kaempferi* (1), *R. macrospalum* (2), and *R. indicum* ‘Ōsakazuki’ (3) and long-lasting flowers *R. kaempferi* ‘Nikkō-misome’ (4), *R. macrosepalum* ‘Kochō-zoroi’ (5), *R. indicum* ‘Chōjyu-hō’ (6), and *R. × hannoense* ‘Amagi-beni-chōjyu’ (7).

To analyze evidence of abnormal mRNA sequences, *AP3/DEF* homologs were isolated from gDNA samples of *R. kaempferi* and all long-lasting flowers (**Figure 5**). For isolation of *AP3/DEF* homolog gDNA sequences, PCR amplifications were performed using 5P-F1, 5P-F2, and P1-R primers, which designed on 5′ upstream region in *R. kaempferi*. Approximately 3.5-, 4-, and 5-kb sized bands were detected from normal flowers, *R. macrosepalum* ‘Kochō-zoroi,’ and *R. indicum* ‘Chōjyu-hō,’ respectively, and over 10-kb sized bands were detected from *R. kaempferi* ‘Nikkō-misome’ and *R.* × *hannoense* ‘Amagi-beni-chōjyu’ (**Figure 5A**). PCR products of 5P-F1 and 5P-F2 primers were used for cloning of *AP3/DEF* homolog in *R. kaempferi*. However, PCR products of either 5P-F1 and/or 5P-F2 primers were used to analyze *AP3/DEF* gDNA sequences in long-lasting flowers, because too large-sized bands were detected from gDNAs of long-lasting flowers. The length of the *RkAP3a* and *RkAP3b* sequences, including the 5′ upstream region, seven exons, and six introns, was 3,772 (DDBJ Acc. No. AB861603) and 3,804 bp (DDBJ Acc. No. AB861604), respectively (**Supplementary Figure S1C**). The sequence analysis in long-lasting flowers predicted full-length sequences of the *AP3/DEF* homolog region from *R. kaempferi* ‘Nikkō-misome,’ *R. macrosepalum* ‘Kochō-zoroi,’ *R. indicum* ‘Chōjyu-hō,’ and *R.* × *hannoense* ‘Amagi-beni-chōjyu’ as approximately 17,102 bp (*RkAP3NM*, DDBJ Acc. No. AB861605), 4,507 bp (*RmAP3KZ*, DDBJ Acc. No. AB861606), 6,887 bp (*RiAP3CH*, DDBJ Acc. No. AB861607), and 17,070 bp (*RhAP3AC*, DDBJ Acc. No. AB861608), respectively (**Figure 5B**).

### Insertion Sequences in Azalea *AP3/DEF* Homolog Are LTR-Retrotransposon or Unknown Sequence

Inserted sequences and regions were revealed by sequence comparison among isolated sequences (**Figure 5B**). The inserted *RkAP3NM* sequence in was approximately 13,488 bp and located in exon 7. This sequence contained both 688-bp long-terminal repeats (LTRs) in the same orientation. The inset in *RhAP3AC* was approximately 13,420 bp and located in exon 1. This sequence contained both 690-bp LTRs in the same orientation. The LTR-retrotransposon in both *RkAP3NM* and *RhAP3AC* had similar sequences, but the inserted sequence in *RkAP3NM* was oriented in the opposite direction from *RhAP3AC*. In addition, the insert in *RiAP3CH* was 3,206 bp and located in exon 2. This insert contained the same 359-bp LTRs on both the left and right sides. The other insert in *RmAP3KZ* was 815 bp and located in exon 1. Although this inserted sequence did not correspond to the LTRs of *RkAP3NM* and *RiAP3CH*, the inserted sequence in *RmAP3KZ* was predicted as an LTR, reflecting the presence of TA and CA sequences, which are start and stop sequences (Kumar and Bennetzen, 1999), respectively, in other LTR-retrotransposons. Compared with part of the primer binding (PBS) site and polypurine tract (PPT) among other transposable elements (TEs) (Smyth et al., 1989; Kumekawa et al., 1999), we proposed that the TE in both *RkAP3NM* and *RhAP3AC* were *Ty1-copia* type and the TE in *RiAP3CH* was a *Ty3-gypsy* type (**Figure 5C**).

Moreover, to confirm the mutant gene status in long-lasting flowers, we searched the gDNA of long-lasting flowers using multiplex-PCR with three primers (**Figure 5B** and **Supplementary Table S1**) designed on exon and putative TE sequences. The PCR product obtained using the EP1 primer set was amplified in normal and *RkAP3NM* samples, and the PCR product obtained using the EP2 primer set was amplified from all of the samples, except *RkAP3NM*. In addition, the PCR products of the other primer sets were amplified from each region containing a TE insertion (**Figure 5D**). The long-lasting flower contained the mutant gene, but not the normal gene, suggesting that the TE insertion likely leads to long-lasting flowers and that the long-lasting flowers likely carry homozygous mutant genes.

### Long-lasting Mutant Was Caused by *ap3/def* Alleles

**FIGURE 6:**
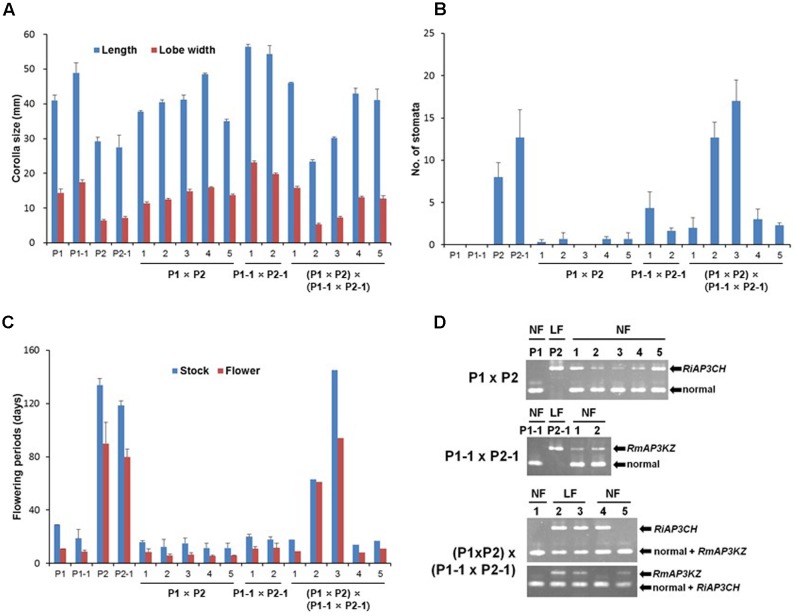
Investigation of floral morphology and mutant gene in progenies resulting from a cross between normal (NF) and long-lasting flowers (LF). **(A–C)** Analysis of floral morphology, such as corolla sizes (mm) **(A)**, number of stomata **(B)**, and flowering periods (days) **(C)**. **(D)** Mutant allele analysis using PCR and mutant gene-specific primers (**Supplementary Table S1**). P1, Kurume hybrid ‘Wakakaede’; P1-1, *R. oldhamii*; P2, *R. indicum* ‘Chōjyu-hō’; P2-1, *R. macroseplaum* ‘Kochō-zoroi’; F1, P1 × P2; F1-1, P1-1 × P2-1; F2, (P1 × P2) × (P1-1 × P2-1).

To determine whether the mutant allele is essential for long-lasting flowers, we investigated floral phenotypes and genotypes using cultivars (*n* = 4) and individual progenies (*n* = 12) through multiplex-PCR with three primers (**Supplementary Table S1**) that were used to distinguish each *ap3/def* mutant allele in evergreen azaleas based on differences in the TE insertion regions (**Figure 6**). All of the F1 progenies resulting from the cross between normal and long-lasting flowers showed normal flowers, whereas F2 progenies showed normal and long-lasting flowers (**Figures 6A–C**). Consistent with the observed phenotypes, normal genes were detected in all of the F1 progenies and one F2 progeny, and only mutant alleles, both *RiAP3CH* and *RmAP3KZ*, were detected from the two F2 progenies with long-lasting flowers, whereas normal and mutant genes, *RiAP3CH* or *RmAP3KZ*, were detected from the F2 progenies with normal flowers (**Figure 6D**). As heterozygous alleles, combination of normal and mutant flowers, in F1 and F2 leads to normal flower development and the homozygous mutant alleles permit long-lasting flower development in F2, we concluded that long-lasting flowers are conferred through homozygous *ap3/def* mutant alleles.

## Discussion

Our investigation of floral morphology and molecular characters in long-lasting flowers showed that the characteristics of evergreen azaleas with flowers, including long-lasting, small-sized, and sepaloid corollas, are unique to these species and are rarely found in other horticultural plants.

We have shown that azalea *AP3/DEF* homolog has functions similar to those of *Antirrhinum DEF* gene in regulation of long-lasting flower development (Schwarz-Sommer et al., 1992). The *Antirrhinum def* mutants have several phenotypes as *chlorantha* and *nicotianoides* flowers that are smaller and their petals show green color indicating sepaloid features. In addition, *gloifera* petals are morphologically indistinguishable from sepals, except that these sepaloid whorl 2 organs are larger than sepals in the whorl 1 and that their position in the mature mutant flower resembles the position of wild type petals of the upper and lower lobes. In analysis of *Antirrhinum GLO* and *DEF* expressions, *DEF* gene is lowly expressed than *GLO* gene expression in *chlorantha* and *nicotianoides* flowers, both genes were not detected from *gloifera* flower buds (Sommer et al., 1990; Schwarz-Sommer et al., 1992; Tröbner et al., 1992). In other plants, an investigation of the relationship between the observed phenotype and *PhDEF* gene expression in the *Petunia hybrid* artificial *phdef* mutants showed that the corolla was converted to sepals in the whorl 2, where *PhDEF* gene expression was found to be significantly reduced (Vandenbussche et al., 2004). When *AP3* homolog expression is repressed in oilseed rape (*Brassica napus*) and poppies (*Papaver somniferum*) flower mutants, petals of the transformed plants are converted to sepaloid petals, which are smaller than normal petals; furthermore, in these mutant plants, stomata grow from the sepaloid petal in the same manner as the sepal (De Byzova et al., 2004; Drea et al., 2007). Also, Hirai et al. (2010) suggested that the reduced expressions of *TGDEFA* and *TGDEFB* are associated in the floral development of the viridiflora tulip (‘White Dream’ and ‘Spring Green’), which is partially composed of sepaloid tepals in the first and second whorl. As case of herbaceous peony (*Paeonia lactiflora*), spontaneous corolla mutant in natural condition was closely associated with selective expression alterations of duplicated *AP3* and *PI* genes, because down-regulation of duplicated B-class genes might result in reduction of *AP3-PI* heterodimer in sepaloid corolla (Gong et al., 2017). Although phenotype is different, *Malus* × *domestica PI* (*MdPI*) mutant, which inserted retrotransposon in natural condition, was weakly expressed in mutant plant; some apples produce parthenocarpic fruit, resulting in LTR-retrotransposon insertion in the *MdPI* MADS transcription factor; Intron 4 of *MdPI* has insertions in ‘Rae Ime,’ and intron 6 has insertions in ‘Spencer Seedless’ and ‘Wellington Bloomless.’ In these mutant cultivars, flowers have no petals or stamens but two whorls of five sepals and an increased number of styles and carpels (Yao et al., 2001), due to insertion into the common MADS transcription factor. In this study, long-lasting flowers have functional stamens (**Figure 6**) and azalea *AP3/DEF* homolog was expressed in floral buds of plant materials, except *R.* × *hannoense* ‘Amagi-beni-chojyu’ in RT-qPCR, but results of semi-qRT-PCR and sequence analysis indicated that the abnormal mRNA sequence of azalea *AP3/DEF* exist in floral buds of all long-lasting cultivars (**Figures 3**, **4**). Moreover, TE or unknown sequences were detected from gDNA sequences in *AP3/DEF* homologs of long-lasting flowers (**Figure 5**). Thus, the loss-of-function of the *AP3/DEF* homolog in long-lasting flowers were caused by the insertion of a retrotransposon or putative LTR sequence, and these inserted sequences might be affected by mRNA splicing of the *AP3/DEF* homolog in long-lasting flowers, because gene expression or the structure encoded proteins can be occurred by mutations due to TE insertions in or near genes (Kumar and Bennetzen, 1999; Lisch, 2013).

The original species of four long-lasting flowers were distributed at different site and the original species have been recorded since 17th century in Japan (Ito, 1984; Yamazaki, 1996). The geographic origin of the azalea species indicates the possibility of independent events of mutation, because azalea cultivars were selected from natural hybrid population and the selected cultivars have been propagated by cutting (Kobayashi, 2013). We have analyzed azalea *AP3/DEF* gDNA sequences of four flower mutants and found insertions in all four cultivars; ‘Nikkō-misome’ and ‘Amagi-beni-chōjyu’ have the nearly same insertion sequence in exon 7 and exon 1, respectively, but ‘Kochō-zoroi’ and ‘Chōjyu-hō’ have different insertion sequence in exon 1 and exon 2, respectively (**Figure 5B**). The different TE insertion regions at same gene are good evidence that *ap3/def* mutant is included in the development of long-lasting flower in these azaleas. In addition, when we analyzed normal and mutant alleles from individual azalea cultivars and its progenies, the non-functional *AP3/DEF* mutant gene was detected from long-lasting flowers but normal allele was not detected (**Figures 5D**, **6**). The results corresponded to petal loss plant in apetalous mutant of Nigella; the mutant was recessive trait that indeed caused by the TE insertion in *AP3-3* gene, and the non-functional *AP3-3* gene was existed as homozygous for petal loss (Zhang et al., 2013). In previous study of azalea, long-lasting flower was shown as recessive trait to normal corollas and was controlled by single gene (Gobara et al., 2017). From this, the results of mutant allele investigation in cultivars and progenies suggest that whereas all long-lasting flowers are homozygous for the *ap3/def* mutant alleles, normal flowers are either heterozygous (normal allele/mutant allele) or homozygous (normal alleles). Despite the phenotypic similarity of *Antirrhinum def* mutants (Sommer et al., 1990), it is still difficult to conclude that azalea *AP3/DEF* homolog was participated to long-lasting flower development. A difference between azalea *ap/def* homolog and *Antirrhinum DEF* mutants is that the azalea long-lasting flowers produce corollas, which have long maintenance period. Phenotypic and molecular evidence show that corollas in long-lasting flowers are related to mutations in azalea mutant allele. The corolla persisting period of long-lasting flower after flowering was long, approximately 100 days (**Figures 1C–E**, **2J**,**6C**). When we analyzed normal and mutant allele from individual azalea cultivars and its progenies, the non-functional azalea *ap3/def* mutant allele was detected from long-lasting flowers but normal allele was not detected (**Figures 5D**, **6D**). The sepaloid corolla of long-lasting flowers may affect flower longevity, because the period of sepal persisting is longer than the period of petal persisting after flowering in evergreen azaleas. Although the maintenance of *PeMADS6*, which might have the function of *GLO/PI*-like genes in *Phalaenopsis* in orchids, transcripts in flower through flowering to senescence corresponds to the long-lasting flower longevity in orchids; transgenic plants overexpressing *PeMADS6* or *PI*, respectively, had a 3.4- or 2.1-fold higher flower longevity than wild-type *Arabidopsis* plants (Tsai et al., 2005), the relationship of *AP3/DEF*-like gene and flower longevity has not been reported. Thus, the results in this study showed that the long-lasting flower would be derived from the sepaloid corolla in long-lasting flowers and would be caused by a mutation of the *AP3/DEF* homolog, although more experiments are needed to clarify mechanism of long flower longevity.

The flower quality of the current long-lasting flowers is a desirable trait (Kobayashi et al., 2010; Kobayashi, 2013). To breed long-lasting trait into commercial azalea cultivars, azalea breeding have attempted. However, the breeding process is slow when traditional crossing methods are used in wood plants because of the long juvenile period required. With the identification of the *AP3/DEF* homolog sequence, genetic transformation could be used to generate high-quality, long-lasting flowers by the down-regulation of *AP3/DEF* homolog expression using RNAi techniques together with transgenic systems developed for azalea cultivars. This study firstly revealed LTR-retrotransposons in azalea cultivars and the TEs into *AP3/DEF* homolog may also be used as a gene base marker for the selection of the long-lasting trait at the juvenile period in evergreen azalea breeding. Collectively, our results increase the current understanding of long-lasting flowers. With regard to the nature of the mutation at azalea *AP3/DEF* homolog, this naturally occurring TE-induced phenotypic change may be especially important for variety flower in horticultural plants, as it affects an essential trait for adaptive plant evolution. From more than 300 years ago, unique mutant flowers have been collected from natural population, reflecting the natural selection and maintenance of TE-induced mutant flowers, and Japanese people have been enjoyed mutant flowers as ornamental plant.

## Author Contributions

Experimental design: NK, AN, and K-SC. Experiments: K-SC and KT. Data analysis: K-SC, AN, and KT. Manuscript preparation: K-SC, KT, AN, and NK. Supervision, funding, and reagents: NK and AN.

## Supplementary Material

The Supplementary Material for this article can be found online at: https://www.frontiersin.org/articles/10.3389/fpls.2017.02239/full#supplementary-material

FIGURE S1 Sequence and structure information for *RkAP3a* and *RkAP3b*. **(A)** The cDNA sequences of *RkAP3a* and *RkAP3b* isolated from *R. kaempferi* are shown in the top and bottom lines, respectively. The dots and boxes indicate sequence identity and different sequences between *RkAP3a* and *RkAP3b* mRNA sequences, respectively. The M and asterisk indicate the start and stop codons, respectively. Primers and directions are indicated with horizontal arrows. The intron positions are indicated with vertical arrows. **(B)** Comparison of the amino acids among AP3, DEF, RkAP3a, and RkAP3b. The box indicates the amino acid sequence of the euAP3 motif. **(C)** The gDNA structure of *RkAP3a* and *RkAP3b*. Dotted lines, bold lines, thin lines, and gray boxes indicate the 5′ upstream region, 5′ and 3′ UTR, introns, and exons, respectively.

TABLE S1 Primers used for the sequence and mutant gene analysis in normal and long-lasting flower types.

## Conflict of Interest Statement

The authors declare that the research was conducted in the absence of any commercial or financial relationships that could be construed as a potential conflict of interest.
