# Supplementary figures and images for: Long-lasting Corolla Cultivars in Japanese Azaleas: A Mutant AP3/DEF Homolog Identified in Traditional Azalea Cultivars from More Than 300 Years Ago
Source: Front Plant Sci. 2018 Jan 9;8:2239. doi: 10.3389/fpls.2017.02239 (PMC5767231; doi:10.3389/fpls.2017.02239)

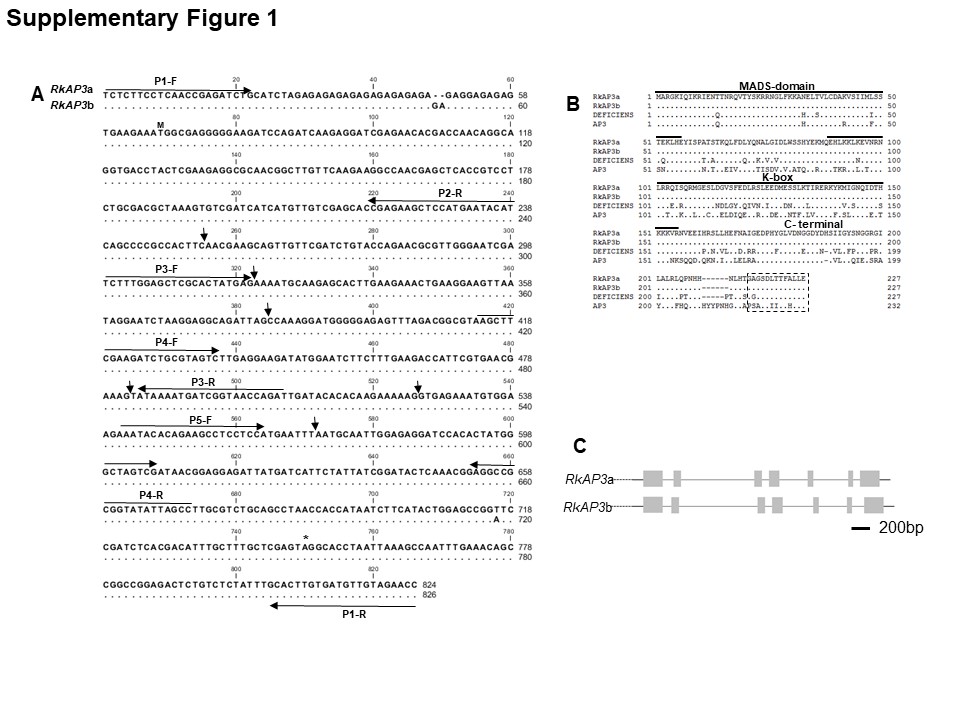

Supplement: FIGURE S1 — Sequence and structure information for RkAP3a and RkAP3b. (A) The cDNA sequences of RkAP3a and RkAP3b isolated from R. kaempferi are shown in the top and bottom lines, respectively. The dots and boxes indicate sequence identity and different sequences between RkAP3a and RkAP3b mRNA sequences, respectively. The M and asterisk indicate the start and stop codons, respectively. Primers and directions are indicated with horizontal arrows. The intron positions are indicated with vertical arrows. (B) Comparison of the amino acids among AP3, DEF, RkAP3a, and RkAP3b. The box indicates the amino acid sequence of the euAP3 motif. (C) The gDNA structure of RkAP3a and RkAP3b. Dotted lines, bold lines, thin lines, and gray boxes indicate the 5′ upstream region, 5′ and 3′ UTR, introns, and exons, respectively. [file Image_1.JPEG]
