# Supplementary material for: Long-lasting Corolla Cultivars in Japanese Azaleas: A Mutant AP3/DEF Homolog Identified in Traditional Azalea Cultivars from More Than 300 Years Ago
Source: Front Plant Sci. 2018 Jan 9;8:2239. doi: 10.3389/fpls.2017.02239 (PMC5767231; doi:10.3389/fpls.2017.02239)
Supplement: TABLE S1 — Primers used for the sequence and mutant gene analysis in normal and long-lasting flower types. [file Table_1.PDF]

Supplementary Table S1. Primers used for the sequence and mutant gene analysis in normal and long-lasting flower types.

Purpose	Name	Sequence (5'-3')
RT-qPCR for expression analysis	RpAP3 -Real-time-F	CACAGAAGCCTCCTCCATGA
	RpAP3 -Real-time-R	TGGTGGTTAGGCTGCATACG
5′RACE-PCR	5RACE-AP3-1	TACAGATCGAACAACTGCTT
	5RACE-AP3-2	GTATTCATGGAGCTTCTCGG
	5RACE-AP3-F2	TCTAAGGAGGCAGATTAGCCA
	5RACE-AP3-P	TTCACGAATGGTCTTCAAAGAAG
Inverse PCR primer for 5’upstream region identity of *AP3/DEF* homologue	AP3-EXON2-F (first forward)	GATCTTTGGAGCTCGCACTAT
	5RACE-AP3-1 (first reverse)	TACAGATCGAACAACTGCTT
	AP3-EXON3-F (second forward)	TAGGAATCTAAGGAGGCAGAT
	5RACE-AP3-2 (second reverse)	GTATTCATGGAGCTTCTCGG
Primers for *AP3/DEF* homologue sequencing	T7	TAATACGACTCACTATAGGG
	M13-reverse	CAGGAAACAGCTATGAC
	AP3-isolate-F	GATGCTAAAGTGTCGATCATC
	AP3-EXON3-F	TAGGAATCTAAGGAGGCAGAT
	5RACE-AP3-F3	GCTTCGAAGATCTGCGTAGTC
	5RACE-AP3-P	TTCACGAATGGTCTTCAAAGAAG
	AP3-PI motif	GGCTAATATACCGCGGCCTC
	AP3-EXON5-R	TGTATCAATCTGGTTACCGATCA
	AP3-INTRON2-R	AGGTATCTGCCATAAACCATA
Retrotransposon of *R. indicum* ‘Chōjyu-hō’	AP3-TYO-INTRON2-F	GGGGTTTTCACTATATCGAGA
	AP3-TYO-R6	TAGTGGAGTCCTTCGGGTTT
	AP3-TYO-R5	AAGTGGTGATTCTGTTGCCG
	AP3-TYO-R4	TTCTGGATCTGTAAAGCTCGT
	AP3-TYO-R3	GAGCTCCAAAGATCGATTCC
Retrotransposons of *R. kaempferi* ‘Nikkō-misome’ and *R. × hannoense* ‘Amagi-beni-chōjyu’	AP3-RETRO(NIKKO)-F1	TTGCCCCCTATTTACTTGAGT
	AP3-RETRO(NIKKO)-R1	GGAGGGCATCTTACTAATTTGG
	AP3-RETRO(NIKKO)-F2	CCCAAGTCAAATTATCCTCTCA
	AP3-RETRO(NIKKO)-R2	CAAGATATGTACGGTCCAGGA
	AP3-RETRO(NIKKO)-F3	TTCCCCAAACAGAGACAATTATT
	AP3-RETRO(NIKKO)-R3	CAGAGAAACCAATGAATATGCC
	AP3-RETRO(NIKKO)-F4	ATCAACAGGAGAAAAACGAGG
	AP3-RETRO(NIKKO)-R4	GCAGAGGTCTTTATGGATATGG
	AP3-RETRO(NIKKO)-F5	GTTGAGGCTCGTGTCTATTGAT
	AP3-RETRO(NIKKO)-R5	CCAGCCTATCTCAAAAGTGTTG
	AP3-RETRO(NIKKO)-F6	CTCTCATTCCTCAAATCACCTC
	AP3-RETRO(NIKKO)-R6	GGGCTGAATGTGTTTTTGAT

Supplementary Table S1. continued

Retrotransposons of *R. kaempferi* 'Nikkō-misome' and *R.* × *hannoense* 'Amagi-beni-chōjyu'	AP3-RETRO(NIKKO)-F7	TTCTGGGCTCTAATTTGGTTC
	AP3-RETRO(NIKKO)-R7	TGGGTAGTTATGGCTTTTGGA
	AP3-RETRO(NIKKO)-F8	CCATCTCATTTTATCTCCAGCTT
	AP3-RETRO(NIKKO)-R8	ACGAGCAAGGTAGAGAACGAT
	AP3-RETRO(NIKKO)-F9	TGAGGCACGTATTCGAAATC
	AP3-RETRO(NIKKO)-R9	AACAGAAAGGACAGTGAGATGC
	AP3-RETRO(AMAGI)-R(2)	TAAATTCCTCATGGCAGTTCC
	AP3-RETRO(AMAGI)-R(3)	GCTGCTTCGTCTTCTGGATTA
Primers for mutant gene analysis in cultivars (Fig. 5D)	EP1-F	GCGAATACCTAGAACGAAGCA
	EP1-R	GAGCTCCAAAGATCGATTCC
	EP2-F	AAATACACAGAAGCCTCCTCCA
	EP2-R	AAGCAAATGTCGTGAGATCG
	AP-R	AAAAGTAGAAGCCTTTGGGC
	KP-R	ATAAAGCGGTTACCAGATCTG
	CP-F	GGGGTTTTCACTATATCGAGA
Primers for ***RiAP3CH*** analysis in progenies (Figure 6D)	AP3-CHOJYU(MAR)-F1	GCGAATACCTAGAACGAAGCA
	AP3-CHOJYU(MAR)-R1	ATGGAGGAGCGTGGATTAAA
	AP3-CHOJYU(MAR)-R2	GGGACCGATACCGCTATTTA
Primers for ***RmAP3KZ*** analysis in progenies (Figure 6D)	AP3-AMAGI(MAR)-F1	GAGAGTGAAGAAATGGCGAG
	AP3-KOCHO(MAR)-R	GGATAACGCCTCTAGAAGGG
	5RACE-AP3-2	GTATTCATGGAGCTTCTCGG
